# Nutritional Status and Nutritional Treatment Are Related to Outcomes and Mortality in Older Adults with Hip Fracture

**DOI:** 10.3390/nu10050555

**Published:** 2018-04-30

**Authors:** Vincenzo Malafarina, Jean-Yves Reginster, Sonia Cabrerizo, Olivier Bruyère, John A. Kanis, J. Alfredo Martinez, M. Angeles Zulet

**Affiliations:** 1 Department of Nutrition, Food Science and Physiology, Faculty of Pharmacy and Nutrition, University of Navarra, 31008 Pamplona, Spain; jalfmtz@unav.es (J.A.M.); mazulet@unav.es (M.A.Z.); 2 Geriatric Department, Complejo Hospitalario de Navarra, 31008 Pamplona, Spain; 3 Department of Public Health, Epidemiology and Health Economics, University of Liège, CHUB23, 4000, Liège, Belgium; jyr.ch@bluewin.ch (J.-Y.R.); olivier.bruyere@ulg.ac.be (O.B.); 4 WHO Collaborating Center for Public Health Aspects of Musculoskeletal Health and Aging, Liège, Belgium; 5 King Saud University, 11692 Riyadh, Kingdom of Saudi Arabia; 6 Dietician Researcher; 08025 Barcelona, Spain, sonia.abril51@gmail.com; 7 Centre for Metabolic Bone Diseases, University of Sheffield Medical School, University of Sheffield, Beech Hill Road, Sheffield S10 2RX, UK; w.j.pontefract@sheffield.ac.uk; 8 Institute for Health and Aging, Catholic University of Australia, Fitzroy VIC 3065 Melbourne, Australia; 9 Navarra Institute for Health Research (IdiSNA), 31008, Pamplona, Spain; 10 CIBERobn, Physiopathology of Obesity and Nutrition, Instituto de Salud Carlos III, 28029 Madrid, Spain; 11 Centre for Nutrition Research, Faculty of Pharmacy and Nutrition, University of Navarra, 31008 Pamplona, Spain; 12 IMDEA Food, Research Institute on Food & Health Sciences, 28049 Madrid, Spain.

**Keywords:** older adults, hip fracture, malnutrition, body mass index, nutritional biomarkers

## Abstract

Malnutrition is very prevalent in geriatric patients with hip fracture. Nevertheless, its importance is not fully recognized. The objective of this paper is to review the impact of malnutrition and of nutritional treatment upon outcomes and mortality in older people with hip fracture. We searched the PubMed database for studies evaluating nutritional aspects in people aged 70 years and over with hip fracture. The total number of studies included in the review was 44, which analyzed 26,281 subjects (73.5% women, 83.6 ± 7.2 years old). Older people with hip fracture presented an inadequate nutrient intake for their requirements, which caused deterioration in their already compromised nutritional status. The prevalence of malnutrition was approximately 18.7% using the Mini-Nutritional Assessment (MNA) (large or short form) as a diagnostic tool, but the prevalence was greater (45.7%) if different criteria were used (such as Body Mass Index (BMI), weight loss, or albumin concentration). Low scores in anthropometric indices were associated with a higher prevalence of complications during hospitalization and with a worse functional recovery. Despite improvements in the treatment of geriatric patients with hip fracture, mortality was still unacceptably high (30% within 1 year and up to 40% within 3 years). Malnutrition was associated with an increase in mortality. Nutritional intervention was cost effective and was associated with an improvement in nutritional status and a greater functional recovery. To conclude, in older people, the prevention of malnutrition and an early nutritional intervention can improve recovery following a hip fracture.

## 1. Introduction

Hip fractures represent a significant health risk for older populations because the incidence of fractures increases notably with age [1].

Hip fractures in geriatric patients have a negative impact on functional status and quality of life, and are associated with high mortality [2,3]. Despite the reduction in pre-surgery hospital stay (surgery performed in the first 24 h, or 48 h after admission, is associated with fewer post-operative complications) [4], and improvements in the management of complications, many patients with hip fracture presented functional deterioration [5]. Identifying the risk factors that predict functional loss after a hip fracture could reduce the costs associated with the need for help resulting from loss of autonomy [6] and institutionalization [7], and could also improve the treatment of post-operative complications. The need for help in order to be able to walk within a patient’s home, Parkinson’s disease, smoking, having suffered delirium in the previous month, having a Body Mass Index (BMI) < 22 kg/m^2^, and age are among the independent risk factors for hip fractures [8]. Poor nutritional status, defined by the Mini Nutritional Assessment (MNA), was associated with a higher risk of fracture at any site [9]. Among risk factors for hip fracture as well as functional loss after the fracture, malnutrition represents an area of great interest, principally because it is a modifiable risk factor. The identification of malnutrition is widely accepted as an appropriate procedure, which may help to give patients better care [10]. This review represents an actualization of the evidence previously published on this topic. The novelty of this review is that we included not only studies with nutritional interventions, but also studies that have assessed the nutritional status in older patients with hip fracture.

The principal objective of this review is to describe how both nutritional status, as revealed by malnutrition biomarkers, influences the clinical evolution and mortality of older people with hip fracture, as well as the impact of nutritional intervention. We therefore structured this paper into four chapters concerning subjects with hip fracture: (1) prevalence of malnutrition and nutritional status aspects (including anthropometry, blood biomarkers, and energy intake), (2) influence upon outcomes and complications, (3) mortality, and (4) effects of nutritional intervention.

## 2. Material and Methods

### 2.1. Data Sources and Search Strategy

A search was carried out on the electronic database MEDLINE for papers published from January 1990 until December 2017. The search strategy is detailed in Supplementary data. The search was restricted to articles in English, Spanish, or Italian. The references of the selected articles were manually revised in the search for eligible articles. Whenever there were studies with multiple publications about the same population, the study with the largest sample was selected, as long as it respected our inclusion criteria.

### 2.2. Inclusion and Exclusion Criteria

We included observational and cohort studies that evaluated the presence of malnutrition (defined by MNA, BMI, albumin concentration, or weight loss), and the influence of malnutrition, as revealed by nutritional biomarkers, on functional recovery, post-operative complications, and mortality in hip fracture patients. We considered as nutritional biomarkers: (1) anthropometric parameters, such as BMI, mid-arm circumference, and triceps skinfold; (2) blood concentrations of total proteins, albumin, and micronutrients such as vitamin D and calcium. We also included controlled clinical trials with nutritional intervention. We defined an intervention as cases where patients received supplements (either orally, by tube, or intravenously) or advice on the characteristics of the diet (by a specialized nurse or dietician). We consider studies (which included only males, only females, or both sexes) carried out in populations with an average age of 70 years or above. Reviews and protocols that did not provide results were excluded.

### 2.3. Data Extraction

The title and abstract of papers compiled from the search were evaluated by two researchers who carried out data extraction. Doubts and queries were discussed and whenever these could not be solved, the opinion of a third reviewer was requested. Studies were grouped according to their main objective. When necessary we contacted the corresponding author to request data that did not appear in the paper.

### 2.4. Quality Assessment

The quality of the selected studies was determined with both the National Institutes of Health (NIH) Quality Assessment tool for Observational Cohort and Cross-Sectional Studies and the Quality Assessment of Controlled Intervention Studies [11]. These tools have been designed to evaluate internal validity and bias risk for both types of observational and intervention studies, and each consists of 14 evaluation criteria. The criteria for observational studies are: aims of the study, sources of bias, sampling, participation rate, study power, data collection methods, and confounding. The criteria for intervention studies are: objective of the study, population characteristics, sampling, selection criteria, sample size justification, exposure measured, timeframe, categories of exposure, independent variables, exposure over the time, dependent variables, blinded, drop-out, and confounding. The criteria were rated as either yes, no, or “other” (i.e., CD, cannot determine; NA, not applicable; NR, not reported). The overall assessment of the studies were classified as “good”, “fair”, or “poor”.

## 3. Results

This review included 44 papers, which totaled 26,281 subjects with a mean age 83.6 ± 7.2 years. The population was mostly female (73.5%). The overall quality of the included studies was rated as fair (Supplementary Tables S1 and S2).

### 3.1. Prevalence of Malnutrition and Nutritional Status Aspects in Hip Fracture Patients

In all of the studies included, malnutrition was identified by a validated nutritional assessment tool. Nevertheless, the prevalence of malnutrition changed according to diagnostic tool used. The prevalence of malnutrition was 18.7% using the MNA (long or short form), but it was greater if other diagnostic criteria were used (BMI, albumin, or weight loss) (45.7%). The prevalence of malnutrition, of risk of malnutrition, and the diagnostic tool used in each study are presented in Table 1.

In this section we included 10 studies that assessed the nutritional status of older people with hip fracture, with a total of 1575 subjects (88.3% female, mean age 79.6 ± 4 years). The design of the studies, the general characteristics of the populations studied, and the main results are presented in Table 2.

Patients with hip fracture present malnutrition, as demonstrated by the presence of low values of the anthropometric indices. Several studies showed that energy intake in older people is smaller than that required and recommended [12,13,14,15]. They also showed that calorie and protein intake are significantly lower in geriatric patients with hip fracture compared to patients without fracture. Both the reduced intake observed in hip fracture patients and the increase of the energy requirement secondary to the inflammatory state lead to weight loss and a reduction in muscle mass and fat tissue indicators, and this hypercatabolism situation may continue up to 4 months after the fracture [16,17,18].

The importance of a good nutritional status was backed up by studies that observed how higher BMI scores were associated with a lower incidence of hip fractures [19]. An interesting and original study showed that patients with intracapsular fractures presented lower BMI scores than patients with intertrochanteric fractures. Almost half of the subjects with intracapsular fractures presented BMI scores lower than 18 kg/m^2^, versus only one-fifth of patients with intertrochanteric fractures [20].

### 3.2. Influence upon Outcomes and Complications

The general characteristics of the studies included in this section can be found in Table 3.

Espaulella et al. showed how after 6 months’ follow-up only slightly over half of the patients subject to follow-up had recovered the functional status they had before the fracture [36]. The MNA was an independent predictor of functional status upon discharge [30], at four and at 12 months [31]. Malnourished patients are more likely to suffer postoperative delirium [32], as well as other post-operative complications such as sepsis [21] and pressure ulcers [37].

Malnutrition is of double importance as it is a risk factor for hip fracture, and in patients with hip fracture it reduces the ability to recover pre-fracture functional capacity. Indeed, malnutrition is a risk factor for fracture, and malnourished older people generally present a worse functional status before the fracture and frequently recover only partially their pre-fracture level of independency in activities of daily living (ADL) following a hip fracture [27]. Conversely, well-nourished older people tend to improve their functional status at discharge after a hip fracture, as revealed by the motor-Functional Independence Measure (FIM) scale [30].

Malnutrition and risk of malnutrition are more prevalent in geriatric patients with a higher comorbidity [38], in addition to being risk factors for complications following hip fracture surgery, such as pressure ulcers [39].

Albumin could be a good blood marker of malnutrition [40]. In this context, Bohl et al. studied a large database (17,651 patients with hip fracture, mean age 84.4 ± 7.2 years) and observed a prevalence of malnutrition of 45.9%, defined as albumin values below 3.5 g/dL prior to surgery [21]. These authors reported that patients with hypoalbuminemia presented a higher prevalence of sepsis (*p* < 0.001), longer hospital stay (*p* < 0.001), and higher prevalence of readmission (*p* = 0.054). The benefits of a good nutritional status were also observed in other studies [18].

### 3.3. Malnutrition and Mortality in Older People with Hip Fractures

In this section we included those studies whose main objective was to assess the impact of malnutrition, as revealed by nutritional biomarkers, on mortality. In addition, we considered studies where a multivariable analysis was carried out and which included malnutrition biomarkers. A summary of the design, characteristics, and main results of the included studies can be found in Table 4. We included five studies, with a total of 2518 patients (71.8% females), mean age 84.3 ± 7.2 years.

Mortality was inversely associated with pre-surgery albumin levels, and patients with hypoalbuminemia had a relative risk of dying of 1.52 (95% Confidence Interval (CI) 1.37–1.70, *p* < 0.001) [21]. Regardless of the tool used to diagnose malnutrition, low values of albumin or BMI or low MNA were associated with an increase in mortality. Albumin concentrations of less than 36 g/L were associated with a 4-year mortality nearly six times greater (Odd-Ratio (OR) 5.85, 95% CI 2.3–16.5) [43]. Furthermore, BMI values of less than 22 kg/m^2^ were associated with an increase of almost seven times the mortality at 1 year, as compared to values higher than 25 kg/m^2^ (Hazard Ratio (HR) 7.25, 95% CI 1.6–33.7) [22]. Studies such as that of Flodin and collaborators confirm the anterior outcome, observing that subjects with a BMI greater than 26 kg/m^2^ had a risk almost three times less of dying after 1 year from the fracture (OR 2.6, 95%CI 1.4–5.0) [44].

Cenzer et al. showed that difficulty preparing meals after hip fracture predicts 1-year mortality (and this predictor factor has the same points as congestive heart failure) [45]. Others factors such as age, male sex, congestive heart failure, and not being able to drive complete the risk stratification scale [45].

Mortality increases progressively after a hip fracture, from an in-hospital mortality of 7%, to 11% in the first 6 months after the fracture, up to 30% in the first year and 40% at 3 years. To highlight the importance of this health problem, we summarized total mortality and the follow-up periods of the included studies in Table 5.

### 3.4. Effects of Nutritional Intervention

In this section we included the studies in which nutritional interventions were carried out. The general characteristics of the populations included, the design, and the main results of the studies included are presented in Table 6.

We included 18 studies, 14 of which were carried out in Europe, one in the USA, one in Australia, and two in Asia, totaling 2248 patients (each study including between 23 and 420 subjects), with an mean age of 81.6 ± 5.4. Five studies were carried out only on women, whereas the rest had mixed samples (66.8% women).

A majority of the studies (*n* 14) used oral nutritional supplements. One was preceded by supplementation with parenteral nutrition. In one study the supplement was administered via naso-gastric tube, and in one other study only dietary advice was used. One study did not specify the type of intervention. The characteristics of the interventions, calories used, protein content, and duration of the treatment are summarized in Table 7.

Regarding duration, in seven studies intervention was maintained during hospital stay, in four studies the duration was ≤3 months, and in two it was up to 6 months. Two of the studies did not specify the duration of treatment.

The results demonstrated that a good compliance in the use of oral supplements was associated with an increase in total energy, protein, and liquid intake during hospital stay [16,17,23,24,48]. This is important because higher nutritional intake was associated with less postoperative complications. This improvement in intake brought on an increase of IGF-1, a decrease of bone loss 1 year after the fracture [50], lesser prevalence and intensity of delirium, and lower production of oxidative stress-derived products [23]. Nutritional supplementation could also lead to a decrease in the incidence and duration of pressure ulcers, as well as delay their onset. Weight loss was found among subjects who received no supplementation (the control group) [25,51,52], probably due to a loss of muscle mass [53,54]. Two recent studies used supplements enriched with Calcium β-Hydroxy-β-Methylbutyrate (CaHMB); these studies observed an improvement in muscle indices in the intervention groups but no improvement in the control groups [54,55].

A multidisciplinary approach is required in order to reduce malnourishment in subjects admitted to hospital [25]. Having a dietician on the team [49] as well as nurses trained in nutrition [25] was associated with an increase in energy, protein, and supplement intake. In addition, a multidisciplinary approach was shown to counteract increases in the incidence of malnutrition after discharge [25]. Furthermore, nutritional intervention was associated with lower short- and long-term mortality rate as well as with an increase in quality of life (as revealed by the EuroQol-5D scale) [25,49]. Nutritional advice for well-nourished patients was associated with better performance in the ADL, and with a better recovery of the ability to walk [28].

## 4. Discussion

Malnutrition is a subject under intense discussion in geriatric research [57,58,59], as it is very prevalent in older people with hip fracture, it negatively influences functional recovery after fracture, it increases healthcare spending, and it is associated with high mortality. It appears that nutritional intervention aids the prevention of complications in geriatric patients with hip fracture. This review is an attempt to summarize existing evidence of these aspects. To our knowledge, this is the first review to assess the nutritional status of older people with hip fracture and how it influences complications and mortality.

Despite the variability in the main objective of the included studies, the results are homogeneous in the evidence that subjects with hip fracture have anthropometric indices indicative of malnutrition (Table 2). In addition, there is evidence that subjects with worse nutritional status have more complications (Table 3) and increased mortality (Table 4). There is a lot of variability in the main objective, as well as in the type of nutritional intervention (dietary advice, use of nutritional supplements) and in the amount of calories used in the included studies (Table 7). Despite this variability in the methods used in the studies, overall nutritional intervention has been shown to reduce complications and avoid weight loss in elderly subjects with hip fracture (Table 6).

The prevalence of malnutrition in older patients with hip fracture is higher than in community-dwelling older adults [60,61]. A further problem was associated with an increase in calorie expenditure, secondary to systemic inflammatory response, without a corresponding intake increase, whereby nutritional intake remained smaller than requirements due to factors such as pain, being bedridden, and reduced mobility [25,49].

A reduction in intake is often observed in older people, causing it to be lower than requirements [57]. These changes in intake have a multi-factor origin, among which the most frequent factors are alterations in sensory organs, loss of teeth, lack of a principal caregiver, and, in some cases, the adverse effects of certain drugs [62]. These intake alterations constitute a well-known geriatric syndrome defined as anorexia of ageing [63]. Calorie and/or protein deficits can contribute to the pathophysiology of fractures, especially through two mechanisms: (1) loss of strength and muscle mass (sarcopenia), which increases the risk of falls; and (2) low bone mineral density (osteoporosis), which reduces the resistance of bones to trauma, increasing the risk of fracture [1].

The observed variability in the parameters of nutritional status in hip fracture patients could be due to the lack of a universal consensus as to the best measure to diagnose protein-energy malnutrition. This lack of universality limits our comparison of the various studies, also making it difficult to carry out a consistent malnutrition diagnosis, which, in certain cases, can delay the clinical decision to prescribe nutritional treatment for these patients.

Despite this, the observed trend is uniform and shows that malnourished older people are at a greater risk of fracture and that the prevalence of malnutrition is high in geriatric patients admitted with hip fracture. Patients with intracapsular fracture usually have low BMI, while patients with trochanteric fracture tend to have high BMI [20,64]. Low BMI is associated with protein deficit (type II nutrients, important for maintaining weight) and type I nutrients are important for bone metabolism. In relation to this, “BMI paradox” is valid in the elderly, in which an increase in fat mass and a decrease of muscle mass are observed, and for this reason falsely high values of BMI can mask the presence of sarcopenia [65]. Despite the important limitations of the prognostic meaning of BMI in the elderly, this remains a fundamental index to assess the nutritional status for its simplicity and repeatability, and most validated nutrition assessment tools include BMI. Recent articles have proposed that the normal cut-off considered by the World Health Organization (WHO) (18.5–20.0 kg/m^2^) should be modified with the values that have been shown to be associated with lower mortality in the elderly (23–29 kg/m^2^) [66]. It would be advisable to complete the nutritional assessment by evaluating the body composition (with dual-energy absorptiometry (DXA) or with bioimpedance analysis) [67,68]. The problem is different if we consider the concentration of albumin for the diagnosis of malnutrition. The blood concentration of albumin may be a good nutritional index if the inflammatory state is taken into account, considering that its concentration does not depend only on nutritional status [40]. On the other hand, albumin has been shown to be a good prognostic index in hospitalized patients [69].

Screening tools such as the Mini-Nutritional Assessment Short-Form (MNA-SF) were able to diagnose a nutritional problem before it manifested through changes in the biochemical markers of malnutrition (such as albumin or total protein) [70]. Factors such as cognitive impairment and disability in the basic activities of daily living (ADL) were associated with lower scores in the MNA-SF [71]. This tool was also shown to be a predictive factor of destination upon discharge following the fracture [72]. Selective deficiencies such as lack of vitamin D are very prevalent in older people [73].

As well as the known effects of this deficiency on bone metabolism, there is a high concentration of vitamin D receptors in muscle tissue [7]. This situation could explain why the lack of this vitamin (scant diet input, little exposure to the sun, and the ability to make vitamin D within the skin declines with age) is so obviously associated with reduced muscle strength and a worse functional status, involving factors that increase the risk of fall and fracture [42].

The high prevalence of malnutrition in people with dementia could be one of the pathophysiological mechanisms for the high risk of falls and fractures, as well as for their poor functional recovery after a fracture [27,37,42,74]. People with dementia suffered an increase in incidence of hip fracture, as dementia is a risk factor for hip fracture [75]. Strategies for the prevention of hip fractures are very important in people with dementia because they present a higher prevalence of complications, higher risk of institutionalization, and worse functional recovery [3]. Moreover, dementia is an independent predictor for mortality [76].

Malnutrition, which is very prevalent in geriatric patients with hip fracture, is associated with the incidence of complications, with length of hospital stay (and thus increase in cost), and with mortality. Hip fracture continues to be a pathology with high mortality. In spite of the achievement of a reduction in the incidence of hip fractures, mortality has not decreased [77]. Intra-hospital mortality of elderly patients with hip fracture (7.4%) is comparable with mortality of elderly patients with heart failure (8%) [78]. The problem is that hip fracture represents an acute potentially preventable disease, for example by implanting exercise programs that have been shown to reduce the risk of falling [79]. It will be necessary to improve the post-surgical treatment to reduce complications and mortality, which at 3 years is almost twice that of patients with heart failure [80,81].

Patients with hip fracture show a state of hypercatabolism secondary to reduced intake, loss of blood, and inflammation, which leads to a reduction in plasma proteins, which are important mechanisms for the defense of oxidative stress. Cell regeneration determines an increase in the production of free radicals at the site of the fracture. Plasma oxidant markers malondialdehyde (MDA) and advanced oxidation protein products (AOPP) were significantly positively, while albumin and total antioxidant capacity (TAC) are significantly negatively associated with the duration of hospitalization.

Several studies observed lower scores in nutritional indices, such as BMI, in geriatric patients who died following a hip fracture, as compared to those who lived [82]. It may be possible to reduce mortality with adequate nutritional intervention [49]. A difficult question to answer is whether nutritional supplementation is indicated for all patients with hip fracture or only for malnourished patients. Supplementation prevented weight loss in both malnourished and well-nourished patients. This association was directly related to the dose administered [51]. A higher protein intake was associated with a lower risk of post-surgery complications [24], and an adequate energy intake reduced the development of complications and was associated with a shorter duration of hospital stay [17].

Therefore, the results of this review support the indications of the European Society of Parenteral and Enteral Nutrition (ESPEN) guidelines, according to which all older adult patients with hip fracture should receive nutritional supplements during hospitalization [83].

## 5. Conclusions

The prevalence of malnutrition is very high in older people and increases further in older people with high comorbidities as well as in geriatric patients. Malnutrition is associated to functional alterations and this can be a cause as well as a consequence of fractures.

Malnutrition prevention could be associated with a reduction in the incidence of fractures, and with a better functional recovery following hip fracture. Fall prevention campaigns as well as advice on healthy and active ageing have contributed to the reduction in the incidence of hip fractures. The inclusion in care plans for geriatric patients with hip fracture of both nutritional assessments and the treatment of malnutrition could contribute to a better functional recovery and a reduction of mortality.

## Figures and Tables

**Table 1:** Prevalence of malnutrition or risk of malnutrition and nutritional screening tool used in the included studies.

**Reference**	**Total** ***n***	**WN** ***n***	**RMN** ***n***	**MN** ***n***	**Cut-Off for Malnutrition**
[21]	17,651	9549	-	8102	Albumin < 3.5 g/dL
[22]	173	49	-	57	BMI < 22 kg/m^2^
[23]	23	9	7	7	BMI ^†^
[20]	96	59	-	37	BMI < 18.5 kg/m^2^
[24]	60	34	-	26	Weight loss ≥ 5% 1 m, or ≥ 10% 6 m, and/or albumin < 2.7 g/dL
[14]	25	11	11	3	Hospital’s own screening tool ^§^
Total of subjects	18,028	9711	18	8232	
Percentage		53.9%		45.7%	
**Reference**	**Total** ***n***	**WN** ***n***	**RMN** ***n***	**MN** ***n***	**Cut-Off for Malnutrition**
[15]	49	18	23	8	MNA ^‡^
[19]	80	38	35	7	MNA
[25]	127	89	36	2	MNA
[17]	50	32	18	0	MNA
[26]	50	7	29	14	MNA
[27]	97	44	37	16	MNA
[28]	162	59	-	103	MNA
[29]	152	87	-	65	MNA
[18]	215	95	95	25	MNA-SF ^¥^
[30]	204	55	98	51	MNA-SF
[31]	594	316	236	42	MNA-SF
[32]	415	152	185	78	MNA-SF
Total of subjects	2195	992	774	411	
Percentage		45.2%	35.3%	18.7%	

^§^ This screening tool is based on changes in dietary intake, weight, and other risk factors (pressure ulcers, presence of infection, period of fasting, and the need for help with eating and drinking); ^†^ Risk of malnutrition cut-off point: Body Mass Index (BMI) between 20 and 22 kg/m^2^; ^‡^ Mini-Nutritional Assessment (MNA) cut-off points: well-nourished ≥ 24 points, at risk for malnutrition at 17–23.5 points, and malnourished at less than 17 points; ^¥^ Mini-Nutritional Assessment-Short Form (MNA-SF) cut-off points: well-nourished 12–14 points, at risk of malnutrition 8–11 points, and malnourished 0–7 points; WN: well-nourished; RMN: risk of malnutrition; MN: malnourished.

**Table 2:** Nutritional status and biomarkers in patients with hip fracture.

Authors Origin Publication Year	Design Aim Setting	*n* (Male/Female) Age, Mean ± SD (Years) BMI (kg/m^2^)	Anthropometry Measurement of Body Composition Biomarkers	(1) Exclusion Criteria (2) Definition of Malnutrition	Main Outcomes
Mansell UK 1990 [33]	Observational Comparison of anthropometric measurements of women with HF, with healthy volunteers in the community (C) and patients admitted to geriatric wards (G)	*n* 663 (0/663) HF 470 Community 103 Geriatric 90	MAC (cm) HF 22.8 ± 0.2 Community 28.6 ± 0.27 Geriatric 25.9 ± 0.41	(1) For healthy female: housebound or wheelchairs (2) NA	Fractured group were older than healthy subjects (*p* < 0.001). HF vs. Community: ↓ MAC ↓ AMA ↓↓ TSF ↓↓ AFA (*p* < 0.001) Significant MAC reduction per year of age: −0.20 ± 0.03 cm/year (HF) −0.15 ± 0.06 cm/year (Community) Significant TSF reduction per year of age: −0.16 ± 0.03 mm/year (HF)
		HF = 77.3 ± 0.3 years Community 72.5 ± 0.5 years Geriatric 79.1 ± 0.8 years	TSF (mm) HF 13.0 ± 0.6 Community 24.7 ± 0.6		
Maffulli UK 1999 [20]	Observational Nutritional differences in patients with intertrochanteric (IT) and intracapsular (IC) fractures	*n* 119 (91/28) IT 17–54 IC 11–37 80.8 ± 9.1 years 21.5 ± 4.1 kg/m^2^	**Intertrochanteric** TSF 11.6 ± 4.5 mm BSF 6.1 ± 4 mm MAC 23.5 ± 3.6 cm **Intracapsular** TSF 10.6 ± 4 mm BSF 5.4 ± 2.4 mm MAC 21.9 ± 3.1cm	(1) Pathologic fracture (2) BMI < 18 kg/m^2^	Malnourished → 45% IC vs. 20% IT (p < 0.001) 19% Overweight or obese → 22% IT vs. 2% IC Complications 15% IC vs. 3% IT (*p* < 0.05) BMI: IC < IT (20.1 ± 3.3 vs. 22.5 ± 4.6 kg/m^2^, *p* < 0.01)
Murphy UK 2000 [15]	Observational Assess the sensitivity and specificity of MNA, and its comparability with other nutritional tools	*n* 49 (0/49) 79.5 ± 9 years 23.7 ± 4.3 kg/m^2^	Albumin 36.9 ± 4.7 g/L	(1) Cognitive impairment (2) MNA	Patients had low mean values for body weight, albumin and transferrin Mean energy intake was below the estimated average requirementMNA < 17: Sensitivity: 27–57% Specificity: 66–100%
Lumbers UK 2001 [12]	Cross-sectional Intake and nutritional status in HF compared to day center attendees (DC)	*n* 125 HF 75 (0/75) DC 50 (0/50) 80.2 ± 7.9 years 25.5 ± 4.8 kg/m^2^	**HF** MAC 27.1 ± 4.3 cm TSF 17 ± 2.7 mm MUAMC 21.4 ± 3.4 cm **Day Centers** MAC 31.3 ± 4.7 cm TSF 18.9 ± 2.8 mm MUAMC 23.3 ± 3.8 cm	(1) Mental function test < 7 (2) NA	HF patients vs. day center attendees have: lower BMI (24.1 ± 4.7 vs. 27.5 ± 4.9 kg/m^2^, *p* < 0.001); lower MUAMC, albumin, proteins and energy intake and higher CRP (*p* < 0.01) Albumin ↔ RCP (*r* = −0.45)
Nematy UK 2006 [14]	Observational Nutritional status and energy intake	*n* 25 (7/18) 85.3 ± 1.5 years 21.9 ± 1.0 kg/m^2^	Albumin 36 ± 2.6 g/L	(1) Pathological fracture or elective surgery (2) Changes in dietary intake, weight loss, pressure sore, infection, and need help for eating	At risk of malnutrition group (*n* 17) had lower BMI and lower energy intake versus well-nourished group (*n* 8) BMI: ARM 19.6 ± 1.1 vs. WN 25 ± 1.5 kg/m^2^ Energy intake: ARM 3602 ± 320 vs. WN 5044 ± 528 kJ/day
Perez Spain 2010 [19]	Observational Prevalence of malnutrition	*n* 80 (24/56) 80.6 ± 6.3 years 27.1 ± 4.4 kg/m^2^	TSF 5.5 ± 2.3 mm BSF 8.1 ± 4.8 mm MAC 26.8 ± 3.9 mm CC 31.9 ± 4 cm	(1) NA (2) MNA	Length of hospital stay: men 15.3 ± 5.8 days; women 14.9 ± 12 days MNA ↔ BMI *r* = 0.6
Perez Spain 2011 [13]	Observational Nutritional status and intake of HF vs. community dwelling study participants	*n* 86 (0/86) HF = 44 Community = 42	**MAC (cm)** HF 27.3 ± 3.2 Community 29.1 ± 4.1	(1) No osteoporotic fractures or major trauma (2) NA	HF has lower BMI, arm and leg circumference than community dwelling (*p* < 0.05) Energy intake (kcal): HF 1417; community dwelling 2052 (*p* < 0.001) Calcium (mg/dL): HF 827; community dwelling 1265 (*p* < 0.001) Vitamin D (μg/dL): HF 1.6; community dwelling: 5.2 (*p* < 0.001)
		**Age** HF = 77.9 ± 4.7 years Community = 76.2 ± 4.6 years			
			**Calf circumference (cm)** HF 32.5 ± 3.6 Community 35.1 ± 4.4		
		**BMI kg/m^2^** HF = 27.6 ± 3.7 Community = 31.3 ± 4.6			
Koren-Hakim Israel 2012 [18]	Retrospective Association of MNA-SF with functional status, comorbidity, and mortality (36 months)	*n* 215 (61/154) 83.5 ± 6.1 years 26.4 ± 4.9 kg/m^2^	WN28.1 ± 4.0 kg/m^2^ ARM 25.5 ± 5.1 kg/m^2^ MN 22.7 ± 3.7 kg/m^2^	(1) Terminal illnesses and multi-trauma (2) MNA	MNA ↔ BMI, ADL, cognitive status, readmission, mortality 36 m, CCI and CIRS-G Independent variables for mortality → Charlson comorbidity index and functional status (ADL)
Villani Germany 2013 [34]	Cross-sectional Evaluate new screening tool for detection cachexia	*n* 71(19/52) 82.2 ± 5.8 years Men 23.9 ± 2.9 kg/m^2^ Women 25.9 ± 3.8 kg/m^2^	M: MAC (cm) 26.7 ± 3.3 TSF (mm) 11.5 ± 4.8 W: MAC (cm) 27.1 ± 3.9 TSF (mm) 16.4 ± 5.4	(1) Pathological fracture or malignancy, residing in residential care (2) NA	Patients with cachexia: 5 new tool 4 (consensus definition) New tool: Sensitivity 75% and specificity 97% Positive predictive value 60%, negative predictive value 99%
Bell Australia 2014 [35]	Prospective Concurrent and predictive validity of malnutrition diagnostic measures	*n* 142 (45/97) 83.5 years	NA	(1) NA (2) MNA-SF < 8 BMI < 18.5 kg/m^2^ ALB < 35 g/L ICD10-AM Geriatrician (subjective clinical assessment)	Malnutrition prevalence with different tools: BMI (12.7%), MNA-SF (27%), ICD10-AM (48.2%), Albumin (53.2%), subjective assessment (55.1%) MNA-SF ↔ ICD10-AM (*r* = 0.3) and BMI (*r* = 0.2) ICD10-AM ↔ subjective assessment (*r* = 0.6) ICD10-AM independent predictor of 4-month mortality (OR 3.6, 95%CI 1.1–11.8)

ADL: activities of daily living; AFA: arm fat area; AMA: arm muscle area; ARM: at risk of malnutrition; BMI: body mass index; BSF: biceps skinfold; CIRS-G: cumulative illness rating scale for geriatrics; CRP: C-reactive protein; HF: hip fracture; ICD10-AM: international classification of disease 10th revision-Australian modification; MAC: mid-arm circumference; MN: malnourished; MNA: Mini Nutritional Assessment; MUAMC: mid-upper arm muscle circumference; TSF: triceps skinfold; WN: well-nourished. . ↓: lower; ↓↓ much lower; ↔: correlation.

**Table 3:** Association of nutritional status, as revealed by nutritional biomarkers, with outcomes and post-operative complications.

Authors Origin	Design Aim	*n* (Male/Female) Age, Mean ± SD (Years)	BMI (kg/m^2^) Biomarkers	Exclusion Criteria MN Definition Tool	Main Outcomes
Formiga Spain 2005 [41]	Prospective observational Relationship between nutritional status and complications	*n* 73 (12/61) 81.5 ± 7.1 years	Cholesterol 4.3 ± 1.1 mmol/L Albumin 30.6 ± 3.6 g/L TLC/mm^3^ 1278 ± 463	Pathological or multiple fractures, terminally ill patients, surgery delayed >48 h or lipid-lowering drug MNA-SF <11	MNA-SF → 11 ± 0.5 MNA-SF not predict → nosocomial infections and pressure ulcers Albumin predict → nosocomial infections ↓ TLC years ↓ Albumin predict → pressure ulcers Barthel index ↔ Charlson comorbidity index *r* = −0.9 (*p* < 0.0001) Length of hospital stay = 16.4 days
Montero Spain 2007 [42]	Prospective cohort Relationship between malnutrition and recovery	*n* 110 (22/88) 81.4 ± 7.3 years	25(OH)vitD 10.8 ± 5.3 ng/ml TLC/ mm^3^ 1545 ± 592 Albumin 32.6 ± 3.8 g/L Prealbumin 15.3 ± 4.7 mg/dL Cholesterol 160.5 ± 40.8 mg/dL Transferrin 195.9 ± 47.1 mg/dL	Pathologic or major trauma fractures Anthropometric and blood biomarkers	38.8% regained pre-fracture functional state Dementia ↔ ↓ functional recovery 25(OH)vit D <10 ng/ml ↔ ↓ pre-fracture functional state, with bedridden (1 year) and with no functional recovery (*p* < 0.05) Factors associated to bedridden (1 year) OR, 95%CI - pre-fracture functional status 10.02, 2.83–35.47 *p* < 0.01 - Caloric malnutrition 9.57 (2.18–42.84) *p* < 0.01 - Protein malnutrition 15.23 (1.36–1.70) *p* < 0.05
Baumgarten USA 2009 [37]	Prospective cohort Identify care settings associated with increased pressure ulcers risk	*n* 658 (152/506) 83.2 ± 6.6 years	23.8 ± 5.1 kg/m^2^	Fractures occurred during hospital stay Subjective Global Assessment (SGA)	Pressure ulcers at baseline ↔ ↑ severe illness, ↑ comorbidity, ↓ nutritional status, ↓cognitive status (*p* < 0.05) Albumin < 30 g/L: 31.5% Length of hospital stay 5.6 ± 2.8 (no pressure ulcers) vs. 6.6 ± 3.8 (pressure ulcers) (*p* < 0.001)
Drevet France 2014 [26]	Prospective observational Protein Energy Malnutrition prevalence	*n* 50 (15/35) 86.1 ± 4.4 years	22.6 ± 4.3 kg/m^2^	Road accident MNA	Prevalence of PEM was 28% (*n* 14) Mean hospital stay: PEM 21.9 ± 16.7 vs. 13.4 ± 6.7 in non-PEM (*p* = 0.012)
Goisser Germany 2015 [27]	Observational Relationship between nutritional status (MNA) and functional and clinical course	*n* 97 (20/77) 84 ± 5 years	NA	Terminal state, cancer-related pathologic fractures, cancer with acute radiation or chemotherapy MNA	Patients at risk for malnutrition and malnourished: - Baseline, ↑ comorbidities ↑ Charlson comorbidity index ↑ pressure ulcers ↓ cognitive status (*p* < 0.05) - All times, ↓ ADL score (*p* < 0.05) - 68% did not regain pre-fracture ADL - 18% did not regain pre-fracture mobility level (*p* = 0.02)
Bohl USA 2017 [21]	Retrospective Association between albumin with death, and postoperative complications	*n* 17,651 (12,595/5056) 84.4 ± 7.2 years	24.6 ± 5.6 kg/m^2^ Albumin 35 ± 5 g/dL	Preoperative serum albumin concentration not available Albumin concentration	18.5% had BMI < 20 kg/m^2^ Patients with hypoalbuminemia had higher rates: - of death (RR 1.52. 95%CI 1.37–1.70. *p* < 0.001) - of sepsis (RR 1.92. 95%CI 1.36–2.72. *p* < 0.001) - of longer legnth of hospital stay, 5.7 ± 4.7 vs. 5.0 ± 3.9 days (*p* < 0.001)
Helminen Finland 2017 [31]	Prospective Prognostic significance of MNA and albumin	*n* 594 (169/425) 84 years	24.9 kg/m^2^ Albumin 33.5 g/L	Pathological or periprosthetic fractures, institutionalization, prefecture inability to walk MNA-SF	All nutritional measures were significantly associated with mortality Being at risk for malnutrition or being malnourished were significantly associated with impaired mobility at 4 months and 1 year
Mazzola Italy 2017 [32]	Prospective If nutritional status predict postoperative delirium	*n* 415 (104/309) 84 ± 6.6 years	NA Albumin 33 ± 5.4 g/L	Nonoperative approach and preoperative delirium MNA-SF	Risk to develop postoperative delirium: - at risk for malnutrition: OR 2.42, 95%CI 1.29–4.53 - malnourished: OR 2.98, 95%CI 1.43–6.19
Inoue Japan 2017 [30]	Prospective Relationship between nutritional status and functional recovery	204 (39/165) 82.7 ± 9.2 years	20.2 ± 2.5 kg/m^2^ Albumin 36 ± 9 g/L	Terminal disease, chronic liver disease, pre-fracture ambulation difficulty, no weight-bearing, discontinued postoperative rehabilitation MNA-SF	Well-nourished had higher motor-FIM score at discharge Motor-FIM at discharge was significant associated with MNA-SF

ADL: activities of daily living; BMI: body mass index; FIM: functional Independence Measure; HF: hip fracture; MNA: Mini Nutritional Assessment; PEM: protein energy malnutrition; OR: odd-ratio; 95%CI: 95% confidence interval. ↔: correlation.

**Table 4:** Relationship between nutritional status and mortality.

Authors Origin Year Design	*n* (Male/Female) Age, Mean ± SD (Years)	BMI kg/m^2^ (Mean ± SD)	Exclusion Criteria	Main Outcomes
Miyanishi Japan 2010 Retrospective [43]	*n* 129 (24/103) 79 years Survivors 78 ± 11 years Non-survivors 81 ± 10 years	21 ± 2.9 (Survivors) 18.9 ± 3.5 (Non Survivors)	NA	Non-survivors have: ↓* BMI, hemoglobin, albumin and ↑* dementia, complications Mortality predictors (4-year mortality): Albumin (<36 g/L) OR = 5.85 and BMI (<18.9 kg/m^2^); OR = 1.16
Schaller Switzerland 2012 Sub-analysis of RCT [22]	*n* 173 (36/137) 84.2 ± 6.7 years	NA	Severe cognitive impairment (MMSE > 15) or delirium	Risk factor for ↑mortality (1-year mortality): MMSE <25 (HR = 5.77, 95%CI: 1.55–21.55) Male sex (HR = 3.55, 95%CI: 1.26–97) BMI <22 vs. >25 (HR = 7.25, 95%CI: 1.61–33.74) Vitamin D per 1ng/ml (HR = 0.93, 95%CI: 0.87–0.998)
Gumieiro Brasil 2013 Prospective [46]	*n* 86 (20/66) 80.2 ± 7.3 years	NA	Pathological fracture	MNA ↔ gait impairment OR = 0.77 (0.66–0.90) *p* = 0.001 ↑ 1 point MNA → ↑* 29% chance of walking MNA ↔ mortality HR = 0.87 (0.76–0.99) *p* = 0.04 ↑ 1 point MNA → ↓* 15% mortality risk
Flodin Sweden 2016 Prospective [44]	*n* 843 (227/616) 82 ± 7 years	22.7 ± 3.8 kg/m^2^	Severe cognitive impairment, admitted from nursing-homes	1-year mortality (*p* = 0.006): BMI > 26 = 6% BMI 22–26 = 18% BMI < 22 = 16% BMI > 26 indicates a higher likelihood of returning to independent living (OR 2.6, 95%CI 1.4–5.0)
Uriz-Otano Spain 2016 Prospective [47]	*n* 430 (97/333) 84.2 ± 7.4 years	NA	Tumor, high impact fracture	3-year mortality: Albumin HR 0.61, 95%CI 0.42–0.90 Predictors of 3-year mortality: Age, HR 1.04, 95%CI 1.01–1.06 Comorbidity, HR 1.19, 95%CI 1.09–1.30 Complications, HR 1.17, 95%CI 1.05–1.31

MMSE: Mini-Mental State Examinatio; RCT: randomized clinical trial; ↓*: significantly less; ↑*: significantly more.

**Table 5:** Total mortality during hospital stay, and at various stages after discharge.

Reference	In-Hospital	<6 Months	1 Year	36 Months	>36 Months	*n*
[18]	6%			36.7%		215
[20]	6%					119
[21]	7.4%					17,651
[22]			27%			173
[27]	15%					97
[29]		7.70%				152
[31]		30%	26%			594
[35]	4.9%	14.8%				142
[36]	4%	21.1%				171
[39]		29.1%	42.40%			420
[41]	10%					73
[42]	6.4%	11.8%	19.4%			110
[43]					48%	129
[45]			27%			857
[46]		12.8%				86
[48]	1.7%	17.9%				57
[49]	11.6%	20.6%				302
						**23,093**
Total mortality (%)	7.4%	20.4%	29.3%	39.4%	48%	

**Table 6:** Nutritional intervention in patients with hip fracture.

Author Year Origin	Design Aim	*n* (Male/Female) Age, Mean ± SD (years) Follow-Up (FU)	BMI kg/m^2^ (Mean ± SD) Measurement of Body Composition	Exclusion Criteria	Results
Schürch [50] 1998 Switzerland	RCT Effects of oral protein supplements on bone metabolism	*n* 82 (8/74) IG 41 CG 41 80.7 ± 7.4 years 6 months	24.3 ± 4.0 kg/m^2^ MAC (cm) 24.1 ± 3.1	Pathologic fracture, fracture caused by severe trauma, history of contralateral hip fracture, severe mental impairment, bone disease, renal failure, and life expectancy < 1 year	IG (at 6m): ↓ rehabilitation stay (42.2 ± 6.6 vs. 53 ± 4.6 days) *p* = 0.018 ↑increase IGF-1 and IgM *p* < 0.05 50% reduction of proximal femur bone loss (1 year)
Espaulella [36] 2000 Spain	RCT Nutritional supplement and functional recovery, complications and mortality	*n* 171 (36/135) IG 85 CG 86 82.6 ± 6.6 years Follow-up: 6 months	25.4 ± 5 kg/m^2^ MAC: 24.6 ± 3.8 cm Albumin: 35 ± 5.5 g/L	Advanced dementia, intravenous nutrition, pathologic fractures, and accidental falls	Patients with ≥1 complication (6 months): IG 44 (55%) CG 57 (70.4%) *p* = 0.04 IG: ↑ increase albumin (3 months and 6 months)
Bruce [51] 2003 Australia	RCT Nutritional supplements and prevention of weight loss and improvement of outcomes	*n* 109 (0/109) IG 50 CG 59 83.9 ± 7.7 years Follow-up: 6 months	22.8 ± 2.6 kg/m^2^ Albumin 38.8 ± 4.1 g/L	BMI < 20 or BMI > 30 kg/m^2^, residents of nursing homes, diseases that influence nutritional intake, diabetes, and fracture due to a major trauma	Weight loss (all patients): At 4 weeks 31.5% > 5% weight loss 20.7% > 7.5% weight loss At 8 weeks 27.4% > 5% weight loss 14.6% > 5% weight loss Fewer weight loss ↔ ↑ number of cane (*p* = 0.019) and ↑duration of supplementation (*p* < 0.05)
Houwing [56] 2003 The Netherlands	RCT Effect of a high-protein supplement on the development of pressure ulcers	*n* 103 (19/84) 81.0 ± 1.1 years	23.9 ± 0.5 kg/m^2^	Terminal care, metastatic hip fracture, insulin-dependent diabetes, renal disease, hepatic disease, BMI > 40 kg/m^2^.	55.3% developed pressure ulcers stage I or II. Incidence of pressure ulcers stage II: supplement 18%, placebo 28% 57% of patients developed pressure ulcers by the second day
Sullivan [48] 2004 USA	RCT Efficacy of enteral nutrition to decrease complications and long-term outcomes	*n* 57 (39/19) IG 27 CG 30 79 ± 7.6 years Follow-up: 6 months	22.1 ± 4.4 kg/m^2^ BSF: 6.4 ± 3.3 mm Albumin: 33.9 ± 4.5 g/L	Pathological fracture, significant trauma to other organ systems, metastatic cancer, cirrhosis of the liver, and organ failure	IG: ↑ intake of total nutrients *p* = 0.012 At discharge: ↑ Albumin: IG 29 ± 5 vs. CG 25 ± 5 g/L *p* = 0.002
Tidermark [53] 2004 Sweden	RCT Effects of nutritional treatment on nutritional and functional status	*n* 60 (0/60) 82.9 ± 5.4 years Follow-up: 12 months	20.4 ± 2.3 kg/m^2^	<70 years, BMI > 24 kg/m^2^, cognitive impairment and institutionalized, dependent to walk, fractures older than 24 h, pathological fractures, rheumatoid arthritis.	Lean body mass decreased in the CG and protein groups, but remained the same in the protein plus nandrolone group. ADL declined only in the CG.
Eneroth [16] 2005 Sweden	RCT Effects of nutritional supplements on nutritional status and intake.	*n* 80 IG 40 (7/33) CG 40 (10/30) 81.4 ± 7.6 years	23.9 ± 3.8 kg/m^2^	Multiple and pathologic fractures, malignant disease, inflammatory joint disease, dementia, depression, acute psychosis, epileptic seizures, insulin-treated diabetes mellitus, heart, kidney, or liver insufficiency	PEM baseline: CG 33%, IG 38% Fluid intake: IG = 1856 ml, CG = 1300ml (*p* < 0.0001) Energy intake during days 1–10: IG = 1296 kcal/day CG = 916 kcal/day (*p* = 0.003) Difference between actual and needed energy intake: IG = −228 kcal/day CG = −783 kcal/day (*p* = 0.0003)
Duncan [49] 2006 UK	RCT Effectiveness of dietetic assistants (DAs) to reduce in-hospital and 4 months mortality.	*n* 302 (0/302) GT 145 GC 157 83.5 years Follow-up: 4 months	NA	Pathologic fracture	Mortality In trauma unit IG 4%, CG 10% (*p* = 0.048) At 4 months IG 13%, CG 23% (*p* = 0.036) - Energy intake = IG 1105; CG 756 kcal/day (*p* < 0.001) - Supplement intake: IG 409; CG123 kcal/day (*p* < 0.001) - MAC change: IG −0.9; CG −1.3 cm (*p* = 0.002) Weight change: IG −0.35; CG −1 kg (*p* = 0.16)
Hommel [39] 2007 Sweden	Quasi-experimental Effects of an improved care intervention in relation to nutritional status and pressure ulcers	*n* 420 IG 210 (70/140) CG 210 (62/148) 81 ± 10.4 years	24.3 ± 4.4 kg/m^2^ MAC 27.7 ± 4.4 cm TSF 14.8 ± 6.8 mm	NA	Length of hospital stay: IG 11.8 ± 7.4 vs. CG 10.8 ± 5.8 days Pressure ulcers: IG 10%; CG 20.5% (*p* = 0.009)
Botella-Carretero [24] 2010 Spain	RCT Effect of perioperative supplements on nutritional status and postop complications	*n* 60 (16/44) IG 30 (6/24) CG 30 (10/20) 83.6 ± 5.8 years	24.4 ± 3.1 kg/m^2^ TSF 11.9 ± 4.1 mm MAC 24.4 ± 3.2 cm MNA 18.6 ± 3.4 Albumin 33 ± 4g/L	Weight loss > 5% in 1 month or weight loss > 10% in 6 months, albumin < 27 g/L, renal failure, hepatic insufficiency, respiratory failure, and any gastrointestinal condition, any nutritional support in the past 6 months	CG: decrease and worse recovery of albumin and prealbumin (*p* = 0.002; *p* = 0.001) IG: ↑ energy and protein intake (*p* = 0.042; *p* < 0.001) ↑ protein intake → ↓ post-operative complications OR = 0.925 (0.869–0.985) (*p* = 0.003)
Fabian [23] 2011 Austria	RCT Effect of nutritional supplement on post-operative oxidative stress and length of hospital stay	*n* 23 (0/23) IG 14 CG 9 83.8 ± 7.4 years Follow-up: 3 weeks	21.2 ± 3.4 kg/m^2^ Albumin 36.6 ± 3.8 g/L	Renal disease, liver failure, severe congestive heart failure, severe pulmonary disease, and any gastrointestinal condition that might preclude the patient from adequate oral nutritional intake	IG ↑ energy and protein intake (*p* < 0.05) Albumin, total protein, and total antioxidant capacity (post-operative): ↓ CG (*p* < 0.05) ↓ IG Advance oxidation protein products and malondialdehyde: in CG levels still elevated during time but not in IG Length of hospital stay: IG 17 ± 4 vs. CG 19 ± 9 days Albumin ↔ CRP and total antioxidant capacity (*p* < 0.05) Length of hospital stay ↔ AOPP and MDA (*p* < 0.01)
Hoekstra [25] 2011 The Netherlands	Prospective Effectiveness of a multidisciplinary intervention on nutritional status	*n* 127 (31/96) IG 61 CG 66 80.3 ± 8.3 years	26.8 ± 4.5 kg/m^2^	Severe dementia, cancer, pathologic fracture, renal and hepatic dysfunction, pacemaker	IG ↑ energy intake protein, vitamin D, zinc, calcium (*p* < 0.01) IG lower reduction of EuroQol-5D (*p* < 0.05) ↓* BMI, BCM, and FM (3 months) (both groups)
Li [28] 2013 Taiwan	Randomized (1 year) Effects of protein-energy malnutrition on the functional recovery	*n* 162 (51/111) IG 80 CG 82 78.2 years	NA	Cognitive impairment, terminally ill	Malnutrition prevalence: IG 60% vs. CG 67% MN → ↓ performance of ADL, IADL, and recovery of walking ability (*p* < 0.05) IG → ↑ performance of ADL, IADL, and recovery of walking ability (*p* < 0.01)
Wyers [29] 2013 The Netherlands	RCT Cost-effectiveness of dietary intervention comprising combined dietetic counseling and ONS	*n* 152 (108/44) IG 73 CG 79 78.5 years	NA	Pathological or periprosthetic fracture, disease of bone metabolism, life expectancy < 1 year, ONS before hospital admission, dementia.	The additional cost of the nutritional intervention was only 3% of the total cost Total cost was not significantly different between both groups Nutritional intervention was likely to be cost effective for weight as the outcome over 3 months
Myint [52] 2013 Hong Kong	RCT Clinical, nutritional and rehabilitation effects of an oral nutritional supplementation	*n* 121 (41/80) IG 61 CG 60 81.3 ± 6.5 years Follow-up: 6 months	20.7 ± 2.9 kg/m^2^ TSF 12.6 ± 5.6 mm MAC 24.3 ± 3 cm Albumin 29.3 ± 4.6 g/L	Tube feeding, unstable medical condition, BMI ≥ 25 kg/m^2^, malignancy, contraindication for high-protein diet, and mentally incapacitated	BMI decrease of 0.25 and 0.003 kg/m^2^ in the ONS group, and 0.72 and 0.49 kg/m^2^ at hospital and follow-up (*p* = 0.012) Length of hospital stay was shortened by 3.8 days in the ONS group (*p* = 0.04) Intake adequate: 67% in the ONS group, 9% in the control group (*p* < 0.001)
Anbar [17] 2014 Israel	RCT Optimization of supplementation by measurement of resting energy requirements and the effect on outcomes	*n* 50 (17/33) IG 22 CG 28 83.1 ± 6.3 years	24.9 ± 3.9 kg/m^2^	Presented to hospital >48 h after the injury, steroids and/or immunosuppression therapy, oncologic disease, multiple fractures, dementia	ONS = 19.6% of total energy IG: ↑ Energy and protein intake (*p* = 0.001) ↓ complications (*p* = 0.012) and infections (*p* = 0.008) ↓ length of hospital stay (*p* = 0.061) In all patients: Energy balance ↔ complications (r = −0.417; *p* = 0.003) and with length of hospital stay (r = −0.282; *p* = 0.049)
Ekinci [55] 2016 Turkey	RCT Effects of CaHMB on wound healing, mobilization, fat-free mass and muscle strength	*n* 62 (0/62) IG 32 CG 30 82.6 ± 7.1 years	22.0 ± 2.4 kg/m^2^	Diabetes, renal and hepatic failure, gastrointestinal intolerance, endocrine pathology, and dementia.	Patients who were mobile on day 30: - IG 81.3% vs. CG 26.7% (*p* = 0.001) Muscle strength on day 30 was higher in IG vs. CG (*p* = 0.026)
Malafarina [54] 2017 Spain	RCT Effects of ONS on muscle mass and nutritional biomarkers	*n* 107 IG 55 CG 52 85.4 ± 6.3 years	25.4 ± 4.9 kg/m^2^ Albumin 3.1 ± 0.4 g/dL	Diabetes, Barthel index <40 prior to the fracture, tumor, pathological or high-impact fractures	BMI and ALM was stable in IG, but decreased in CG. ONS (*p* = 0.006), function ambulation categories prior to the fracture (*p* = 0.007) and Barthel index prior to the fracture (*p* = 0.007) are protective for loss of ALM

ALM = appendicular lean mass; AOPP: advanced oxidation protein products; BCM: Body Cellular Mass; BMI = body mass index; BSF = biceps skinfold thickness; CG = control group; FM: fatt mass; IADL: instrumental activities o daily living; IGF-1 = insulin-like growth factor; HS = handgrip strength; IG = intervention group; MAC = mid-arm circumference; MNA = Mini Nutritional Assessment; ONS = oral nutritional supplement; RCT = randomized controlled trial; TSF = triceps skin fold thickness; ↓* = significantly less; ↑* = significantly more; ↔ = significant association.

**Table 7:** Characteristics of the nutritional intervention and composition of the nutritional supplementation used in the included studies.

Author Year Origin	Type of Supplement Administration Method	kcal	Nutritional Composition	Treatment Duration Adherence Rate (%)	Control Group
Schürch [50] 1998 Switzerland	Oral liquid supplement; single oral dose of vit D_3_ 200.000 UI Ca: 550 mg/day	250 kcal/day	20 g protein, 3.1 g lipid, 35.7 g carbohydrates, 90% milk proteins	5 days a week for 6 months AR: IG 73% CG 80%	Placebo: 54.5 g carbohydrates Single oral dose of vitamin D 200.000 UI Calcium: 550 mg/day
Espaulella [36] 2000 Spain	Oral liquid supplement 200 mL	149 kcal	20 g protein, 800 mg calcium, 25 IU vitamin D3	60 days AR: IG 94.1% CG 94.2%	Placebo 200 mL, 155 kcal; mainly carbohydrates
Bruce [51] 2003 Australia	Oral liquid supplement (235 mL/day)	352 kcal	17.6 g protein, 11.8 g fat, 44.2 g carbohydrate, vitamins and minerals	28 days after surgery	Hospital diet only
Houwing [56] 2003 The Netherlands	Oral liquid supplement (400 mL/day)	500 kcal	40 g protein	Immediately postoperatively during 4 weeks or until discharge AR: 75% of patients consumed >75% of daily dose	Non-caloric placebo supplement
Sullivan [48] 2004 USA	Standard care + post-operative nightly via enteral feeding tube: 1375 mL (125 mL/h) over 11 h	1031 kcal	85.8 g protein	When volitional intake exceeded 90% of estimated requirements for 3 consecutive days or was discharged: mean 15.8 ± 16.4 days AR: 83.3%	Standard care
Tidermark [53] 2004 Sweden	PR: protein-rich liquid supplement (200 mL/day) PR-N: PR + nandrolone decanoate injections (every third week) 1 g of calcium + 400 IE vitamin D3	200 kcal/day nandrolone: 25 mg intramuscular injection	20 g protein	6 months	Standard treatment 1 g of calcium + 400 IE vitamin D3
Eneroth [16] 2005 Sweden	Hospital diet + intravenous nutrition (1 l/day) followed by 400 mL/day oral supplement	Oral supplement 400 kcal/day	IV: amino acids, fat, carbohydrate, and electrolytes	3 days → IV 7 days → oral	Hospital diet only
Duncan [49] 2006 UK	NA	Mean supplement: 409 kcal/day	NA	NA	Mean standard supplement: 123 kcal/day
Hommel [39] 2007 Sweden	Oral nutritional supplement twice a day	125 kcal/100 mL enriched with arginine, zinc, vitamins A, B, C, and E, selenium, and carotenoids	NA	From post-surgery to discharge	NA
Botella-Carretero [24] 2010 Spain	Oral nutritional supplement intake (2 × 200 mL/day)	400 kcal/day	40 g protein/day	From admission until discharge AR 52.2 ± 12.1%	Control group: no supplement
Fabian [23] 2011 Austria	Oral liquid supplement	Supplements were administered when intake of energy < 20 kcal and/or protein < 1 g/kg body weight/day	40% protein, 41% carbohydrate, 19% fat, vitamins and minerals	From post-surgery to discharge	Standard medical treatment
Hoekstra [25] 2011 The Netherlands	Nurse and doctor encouraged and motivated patients to eat and drink; if MNA < 24, dietician consulted with the patient	NA	NA	NA	Standard nutritional care
Wyers [29] 2013 The Netherlands	Oral liquid nutritional supplement (500 mL/day) Dietetic counseling	500 kcal	40 g protein	Started during hospital admission and continued until 3 months after surgery	Usual care ONS on demand: 13% received ONS and 23% received dietetic counseling
Myint [52] 2013 Hong Kong	Oral liquid nutritional supplement (240 mL twice daily) 1.2 g of calcium + 800–1000 IU vitamin D	500 kcal	18–24 g protein	Started within 3 days after admission until discharge or 28 days AR = 77.7%	NA 1.2 g of calcium + 800–1000 IU vitamin D
Anbar [17] 2014 Israel	Standard ONS (237 mL) or diabetic ONS (237 mL) Patients received the difference between intake and measured energy expenditure	355 kcal 237 kcal	13.5 g protein 9.9 g protein	Started 24 h after surgery AR = 100%	Usual hospital diet = 1800 kcal, 80 g protein
Ekinci [55] 2016Turkey	Oral liquid nutritional supplement (220 mL twice daily)	NA	36 g protein 3 g CaHMB 1000 IU vitamin D	30 days	Usual hospital diet: 1900 kcal, 76 g protein, 63 g fat
Malafarina [54] 2017 Spain	Oral liquid nutritional supplement (220 mL twice daily)	660 kcal	60 g protein 4.6 g CaHMB 1500 IU vitamin D	During hospital admission, until discharge Mean treatment duration: 42.3 ± 20.9 days AR = all of the subjects took more than 80%	Usual hospital diet: 1500 kcal, 87 g protein, 59 g fat

## Supplementary Materials

The following are available online at http://www.mdpi.com/2072-6643/10/5/555/s1, Table S1: Results of quality assessment of the observational included studies, Table S2: Results of quality assessment of included intervention studies.
