# Supplementary material for: Nutritional Status and Nutritional Treatment Are Related to Outcomes and Mortality in Older Adults with Hip Fracture
Source: Nutrients. 2018 Apr 30;10(5):555. doi: 10.3390/nu10050555 (PMC5986435; doi:10.3390/nu10050555)
Supplement: Supplementary file 1 [file nutrients-10-00555-s001.pdf]

Supplementary Table S1. Results of quality assessment of the observational included studies

Year	Author	CK1	CK2	CK3	CK4	CK5	CK6	CK7	CK8	CK9	CK10	CK11	CK12	CK13	CK14	Quality
1990	Mansel	N	N	Y	NR	NR	N	N	NA	NA	NA	Y	NA	Y	N	Poor
1999	Maffulli	N	Y	Y	Y	NR	N	Y	N	N	NA	Y	NR	Y	N	Poor
2000	Murphy	Y	N	NR	NR	N	N	N	NA	N	NA	N	NR	Y	N	Poor
2001	Lumbers	Y	N	NR	Y	Y	N	Y	NA	NA	NA	Y	NR	Y	N	Poor
2006	Nematy	N	N	NR	NR	N	N	N	NA	NA	NA	Y	NR	NA	N	Poor
2010	Perez	Y	Y	NR	Y	N	N	N	NA	NA	NA	Y	NR	NA	N	Poor
2011	Perez	Y	Y	NR	Y	N	N	N	NA	NA	NA	Y	NR	NA	N	Poor
2012	Korem-Hakim	Y	Y	Y	Y	N	N	Y	NA	Y	NA	Y	Y	Y	N	Good
2013	Villani	Y	N	NR	N	N	N	N	NA	N	NA	Y	NR	NA	N	Poor
2014	Bell	Y	Y	Y	Y	N	N	Y	NA	N	NA	Y	NR	NA	Y	Fair
2005	Formiga	Y	Y	NR	Y	N	Y	Y	NA	Y	NA	N	NR	NA	N	Poor
2007	Montero	Y	Y	Y	Y	Y	Y	Y	NA	Y	NA	N	NR	Y	Y	Good
2009	Baumgarten	Y	N	NR	N	N	Y	Y	NA	Y	NA	Y	NR	NR	N	Poor
2010	Miyanishi	Y	N	Y	Y	N	Y	Y	NA	Y	NA	Y	NR	Y	N	Good
2011	Garcia Casanova	Y	Y	Y	Y	N	Y	Y	NA	N	NA	Y	N	N	N	Fair
2012	Schaller	Y	Y	Y	Y	N	Y	Y	NA	Y	NA	Y	NR	Y	Y	Good
2013	Gumieiro	Y	Y	NR	Y	Y	Y	Y	NA	Y	NA	Y	NR	Y	Y	Good
2014	Drevet	Y	N	N	Y	N	Y	Y	NA	Y	NA	N	N	N	N	Poor
2015	Goisser	Y	Y	Y	Y	N	Y	Y	NA	Y	NA	Y	NR	Y	N	Good
2016	Cenzer	Y	Y	Y	Y	NA	Y	Y	NA	Y	NA	Y	N	Y	Y	Good
2017	Bohl	Y	Y	Y	Y	N	Y	Y	NA	Y	NA	Y	NR	Y	Y	Good
2017	Helminen	Y	Y	Y	Y	N	Y	Y	NA	Y	NA	Y	NR	Y	Y	Good
2017	Mazzola	Y	Y	Y	Y	N	Y	Y	NA	Y	NA	Y	NR	Y	Y	Good
2017	Inoue	Y	N	Y	Y	N	Y	Y	NA	Y	NA	Y	NR	Y	N	Good
2016	Flodin	Y	N	Y	Y	N	Y	Y	NA	Y	NA	Y	NR	Y	Y	Good
2016	Uriz	Y	Y	Y	Y	N	Y	Y	NA	Y	NA	Y	NR	Y	Y	Good

Quality of included studies was assessed using the National Institutes of Health (NIH) Quality Assessment tool for Observational Cohort and Cross-Sectional Studies (https://www.nhlbi.nih.gov/health-pro/guidelines/in-develop/cardiovascular-risk-reduction/tools/cohort). CK 1. Was the research question or objective in this paper clearly stated? CK 2. Was the study population clearly specified and defined? CK 3. Was the participation rate of eligible persons at least 50%? CK 4. Were all the subjects selected or recruited from the same or similar populations (including the same time period)? Were inclusion and exclusion criteria for being in the study prespecified and applied uniformly to all participants? CK 5. Was a sample size justification, power description, or variance and effect estimates provided? CK 6. For the analyses in this paper, were the exposure(s) of interest measured prior to the outcome(s) being measured? CK 7. Was the timeframe sufficient so that one could reasonably expect to see an association between exposure and outcome if it existed? CK 8. For exposures that can vary in amount or level, did the study examine different levels of the exposure as related to the outcome (e.g., categories of exposure, or exposure measured as continuous variable)? CK 9. Were the exposure measures (independent variables) clearly defined, valid, reliable, and implemented consistently across all study participants? CK 10. Was the exposure(s) assessed more than once over time? CK 11. Were the outcome measures (dependent variables) clearly defined, valid, reliable, and implemented consistently across all study participants? CK 12. Were the outcome assessors blinded to the exposure status of participants? CK 13. Was loss to follow-up after baseline 20% or less? CK 14. Were key potential confounding variables measured and adjusted statistically for their impact on the relationship between exposure(s) and outcome(s)? CK, check list; CD, cannot be determined; NA, not applicable; NR, not reported; N, no; Y, yes.

Supplementary Table S2. Results of quality assessment of included intervention studies

Year	Author	CK1	CK2	CK3	CK4	CK5	CK6	CK7	CK8	CK9	CK10	CK11	CK12	CK13	CK14	Quality
1998	Schürch	Y	Y	NR	Y	NR	Y	N	Y	NR	NR	Y	NR	NR	N	Poor
2000	Espaulella	Y	Y	Y	Y	NR	Y	Y	N	NR	NR	Y	N	N	N	Fair
2003	Bruce	N	N	N	N	N	Y	NR	NR	NR	NR	Y	N	N	NR	Good
2003	Houwing	Y	NR	NR	Y	NR	Y	Y	Y	N	NR	Y	N	Y	Y	Good
2004	Tidermark	Y	Y	Y	N	NR	Y	Y	Y	Y	Y	Y	NR	N	N	Good
2005	Eneroth	Y	Y	Y	N	N	N	Y	Y	NR	NA	Y	NR	N	Y	Fair
2006	Duncan	Y	NR	NR	N	NR	Y	NR	NR	NR	NA	Y	Y	N	N	Poor
2007	Hommel	N	NA	NA	N	N	N	NR	NR	N	NA	Y	N	N	NA	Poor
2010	Botella Carretero	Y	Y	Y	N	N	Y	Y	Y	N	NA	Y	Y	N	NA	Good
2011	Fabian	Y	NR	NR	N	NR	NR	NR	NR	NR	Y	Y	N	N	N	Poor
2011	Hoekstra	N	NA	NA	N	N	Y	Y	Y	NR	Y	Y	N	N	N	Poor
2013	Li	Y	NR	NR	N	NR	Y	N	Y	NR	NR	Y	NR	N	Y	Poor
2013	Wyers	Y	Y	N	N	NR	Y	Y	Y	NR	NA	Y	N	N	N	Poor
2013	Myint	Y	Y	Y	N	NR	Y	Y	Y	Y	Y	Y	Y	N	N	Good
2014	Anbar	Y	Y	Y	N	NR	Y	Y	Y	NR	NR	Y	Y	N	NA	Good
2016	Ekinci	Y	Y	N	N	N	Y	Y	Y	NR	Y	Y	Y	N	N	Good
2017	Malafarina	Y	Y	N	N	N	Y	Y	Y	Y	Y	Y	N	N	N	Good

Quality of included studies was assessed using the National Institutes of Health (NIH) Quality Assessment of Controlled Intervention Studies (https://www.nhlbi.nih.gov/health-topics/study-quality-assessment-tools). **CK 1.** Was the study described as randomized, a randomized trial, a randomized clinical trial, or an RCT? **CK 2.** Was the method of randomization adequate (i.e., use of randomly generated assignment)? **CK 3.** Was the treatment allocation concealed (so that assignments could not be predicted)? **CK 4.** Were study participants and providers blinded to treatment group assignment? **CK 5.** Were the people assessing the outcomes blinded to the participants' group assignments? **CK 6.** Were the groups similar at baseline on important characteristics that could affect outcomes (e.g., demographics, risk factors, co-morbid conditions)? **CK 7.** Was the overall drop-out rate from the study at endpoint 20% or lower of the number allocated to treatment? **CK 8.** Was the differential drop-out rate (between treatment groups) at endpoint 15 percentage points or lower? **CK 9.** Was there high adherence to the intervention protocols for each treatment group? **CK 10.** Were other interventions avoided or similar in the groups

(e.g., similar background treatments)? **CK 11.** Were outcomes assessed using valid and reliable measures, implemented consistently across all study participants? **CK 12.** Did the authors report that the sample size was sufficiently large to be able to detect a difference in the main outcome between groups with at least 80% power? **CK 13.** Were outcomes reported or subgroups analyzed prespecified (i.e., identified before analyses were conducted)? **CK 14.** Were all randomized participants analyzed in the group to which they were originally assigned, i.e., did they use an intention-to-treat analysis?

Search strategy details

The following term associations were used: older adults/people, elderly, hip fracture/femur, nutrition, nutritional status/biomarkers/intervention/screening tool, risk of/malnutrition/diagnosis/albumin/protein, malnutrition, body mass index, supplement/supplementation, results, outcomes, effects, pressure ulcers, complication, and mortality. We limited the search to articles that had the keywords in their title or abstract.
